# 1293. *In vitro* Activity of Ceftibuten in Combination with VNRX-5236 against Clinical Isolates of Enterobacterales from Urinary Tract Infections Collected in 2018-2020

**DOI:** 10.1093/ofid/ofab466.1485

**Published:** 2021-12-04

**Authors:** Meredith Hackel, Mark G G Wise, Daniel F Sahm

**Affiliations:** 1 IHMA, Inc., Schaumburg, Illinois; 2 IHMA, Schaumburg, Illinois

## Abstract

**Background:**

Increasing resistance among agents commonly prescribed to treat urinary tract infections indicate that new oral agents are urgently needed. Ceftibuten in combination with VNRX-7145 is under development as an oral treatment for complicated urinary tract infections caused by serine β-lactamase-producing Enterobacterales, including isolates carrying ESBLs and carbapenemases. *In vivo*, VNRX-7145 (VNRX-5236 etzadroxil) is cleaved into to the active inhibitor, VNRX-5236. This study assessed the *in vitro* activity of ceftibuten/VNRX-5236 against 592 isolates of Enterobacterales from urinary tract infections (UTIs) from a 2018-2020 global culture collection.

**Methods:**

MICs of ceftibuten with VNRX-5236 fixed at 4 µg/mL and comparators were determined following CLSI M07-A11 guidelines against 592 Enterobacterales. Isolates were from community and hospital UTI infections collected from 133 sites in 31 countries in 2018-2020. Resistant phenotypes were based on 2021 CLSI breakpoints.

**Results:**

A substantial percentage of isolates were non-susceptible to extended-spectrum β-lactams, levofloxacin (LVX), trimethoprim-sulfamethoxazole (SXT), and amoxicillin-clavulanate (AMC) (Table). The addition of VNRX-5236 reduced ceftibuten MIC_90_ values by ≥8-fold to ≥128-fold, depending on the resistant subset. Ceftibuten/VNRX-5236 had potent activity against all Enterobacterales, with MIC_50/90_ values of 0.06/0.25 µg/mL and 98.3% inhibited at ≤2 µg/mL. Ceftibuten/VNRX-5236 maintained activity against resistant subsets (MIC_90_ range, 0.5 to 2 µg/mL; 91.5% to 97.1% inhibited at ≤2 µg/mL), including serine carbapenemase-positive isolates (MIC_90_ 0.5 µg/mL; 100% inhibited at ≤1 µg/mL). Ceftibuten/VNRX-5236 *in vitro* potency was similar to that of newer parenteral and investigational oral therapies.

Results Table

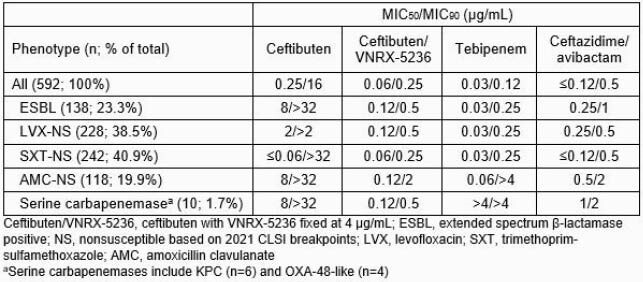

**Conclusion:**

Ceftibuten/VNRX-5236 exhibited promising *in vitro* activity against recent Enterobacterales from UTIs, and may have potential as an oral treatment option for complicated urinary tract infections, including those caused by serine β-lactamase-expressing Enterobacterales (ESBL, KPC, OXA-48/OXA-48-like) for which there are currently few oral treatment options available.

**Disclosures:**

**Meredith Hackel, PhD MPH**, **IHMA** (Employee)**Pfizer, Inc.** (Independent Contractor) **Mark G G. Wise, PhD**, **IHMA** (Employee)**Pfizer, Inc.** (Independent Contractor) **Daniel F. Sahm, PhD**, **IHMA** (Employee)**Pfizer, Inc.** (Independent Contractor)

